# Vaccination willingness in association with personality traits in patients with multiple sclerosis in the course of SARS-CoV-2 pandemic

**DOI:** 10.1038/s41598-022-18912-3

**Published:** 2022-09-07

**Authors:** Felicita Heidler, Julia Baldt, Niklas Frahm, Silvan Elias Langhorst, Pegah Mashhadiakbar, Barbara Streckenbach, Katja Burian, Uwe Klaus Zettl, Jörg Richter

**Affiliations:** 1 Department of Neurology, Ecumenic Hainich Hospital gGmbH, Pfafferode 102, 99974 Mühlhausen, Germany; 2 Department of Neurology, Neuroimmunology Section, University Medical Centre of Rostock, Gehlsheimer Str. 20, 18147 Rostock, Germany; 3 Faculty of Health Sciences, University of Hull, Hull, UK; 4 Durham Law School, Durham University, Durham, UK

**Keywords:** Neuroscience, Psychology, Health care, Neurology

## Abstract

Vaccination is a key strategy for controlling the SARS-CoV-2 pandemic. Acceptance of SARS-CoV-2 vaccines by chronically ill patients, such as multiple sclerosis (MS) patients, plays an important role in prevention of complicated disease course. This longitudinal, prospective, multi-centre-study of German MS-patients aimed to detect socio-demographic, clinical, or psychological determinants of attitudes towards standard vaccines, SARS-CoV-2 vaccines, and governmental measures before/during the pandemic. Exactly 404 MS-patients were investigated by standardized questionnaires and structured interviews on socio-demographic, clinical-neurological, and psychological characteristics, vaccination status, and vaccination from June 2019. Data on SARS-CoV-2 vaccination willingness were collected in two follow-up assessments (1st: June to July 2020, before SARS-CoV-2 vaccine availability, N = 200; 2nd: March to May 2021, after SARS-CoV-2 vaccine availability, N = 157). Age, sex, MS course type, depression, and personality characteristics (Extraversion, Novelty seeking, Self-directedness, and Cooperativeness) were significantly associated with vaccination willingness. Although the majority of MS-patients showed SARS-CoV-2 vaccination willingness at both follow-ups (1st: 60%, 2nd: 61%), a substantial proportion had concerns and were undecided or opposed to vaccination. Socio-demographic variables like age and sex, psychopathological status, and various personality characteristics might influence vaccination willingness and should be considered when discussing with MS-patients about SARS-CoV-2 vaccination.

## Introduction

Since the beginning of the SARS-CoV-2 pandemic in 2020, there are more than 600 million confirmed cases of severe acute respiratory syndrome coronavirus 2 (SARS-CoV-2) infections, including 6.5 million deaths^1^. Vaccination represents the most important intervention in the pandemic management^2–4^. Therefore, it is essential to achieve a major vaccination acceptance among the population^5–8^. The willingness to get a SARS-CoV-2 vaccine is considerably varying in the general population. For instance, the vaccination willingness was 55.8% among English residents regarding a future vaccine^9^ and 69% in U.S. American residents^10^. In January 2021, 60.1% of the 552 Thuringian participants of a German COVID-19 Snapshot Monitoring (COSMO) study indicated the willingness to receive the SARS-CoV-2 vaccination, while 12.0% were undecided and 27.9% did not or less tend to vaccination^11^. In another German study from January 2021, 1779 adults from the general population were online-surveyed shortly after the first SARS-CoV-2 vaccines were approved. The majority of study participants (78%) stated that they would like to become vaccinated or would rather become vaccinated, while 11% were undecided and 11% stated no or rather no willingness to be vaccinated^12^. This study revealed a positive association of anxiety due to COVID-19 as well as health-related fears with the acceptance of SARS-CoV-2 vaccines^12^.

A high level of SARS-CoV-2 vaccination willingness is especially important for people suffering from autoimmune diseases (AI). These patients have a higher susceptibility to infection which can be further increased by immunomodulatory treatments^13–15^. The overall estimated prevalence of AIs varied between 3 and 5%^16^. Multiple Sclerosis (MS) is the most common AI of the central nervous system in young adults with a wide range of neurological deficits such as hyposensibility, dysaesthesia, optic neuritis, paresis, spasticity, gastrointestinal as well as bladder disturbances, fatigue, depression, and neuropsychological deficits^17–22^. The general flu jab rate among German MS-patients was 19% before the SARS-CoV-2 pandemic^23^. A survey among MS-patients living in the U.S.A. between April and May 2020 reported a vaccination willingness regarding SARS-CoV-2 vaccines of 66.0%^24^. The willingness to receive a SARS-CoV-2 vaccination among Portuguese MS-patients was over 80.9% (35.2% definitely willing and 45.7% probably willing) in December 2020/January 2021^25^.

There are several factors which might influence the vaccination willingness of MS-patients during the SARS-CoV-2 pandemic. On the one hand, the increased infection risk of immunocompromised patients and the perceived effectiveness of vaccines, especially of SARS-CoV-2 vaccines, could shape attitudes in favour of a willingness to get vaccinated^24–28^. On the other hand, many MS-patients worry about worsening disease progression and increased disease activity after general vaccination, which might lower vaccination willingness^29^. Another issue that might play an important role in vaccination behaviour of MS-patients in the pandemic and that could lead to a vaccination hesitancy is the safety of the new vector and mRNA vaccines against SARS-CoV-2^9,10,30^. For example, the use of the SARS-CoV-2 vaccine ChAdOx1 nCoV-19 (AZD1222) from Oxford-AstraZeneca (Vaxzevria®) was suspended in some European countries due to the occurrence of thromboembolic events in vaccinated people^31^. Such developments can easily cause insecurity, fear, and distrust in governmental recommendations. All these considerations raise the question if MS-patients' willingness to get vaccinated is solely based on weighing facts and data, or if personality traits also play a decisive role, since personality characteristics represent relatively stable behavioural, emotional, and cognitive phenomena^32^.

It is very important to understand factors that influence the willingness to get vaccinated against SARS-CoV-2. A successful vaccination program has enormous importance on world health and economic status^30^. Therefore, the aim of the present study is to: (1) assess willingness to get a SARS-CoV-2 vaccine and attitudes towards recommended standard vaccinations before as well as during SARS CoV-2 pandemic among German MS-patients; (2) determine socio-demographic, clinical, psychosocial, and personality traits as patient characteristics which might be associated with the general and the SARS-CoV-2 vaccination willingness.

## Methods

### Study design

**Figure 1:**
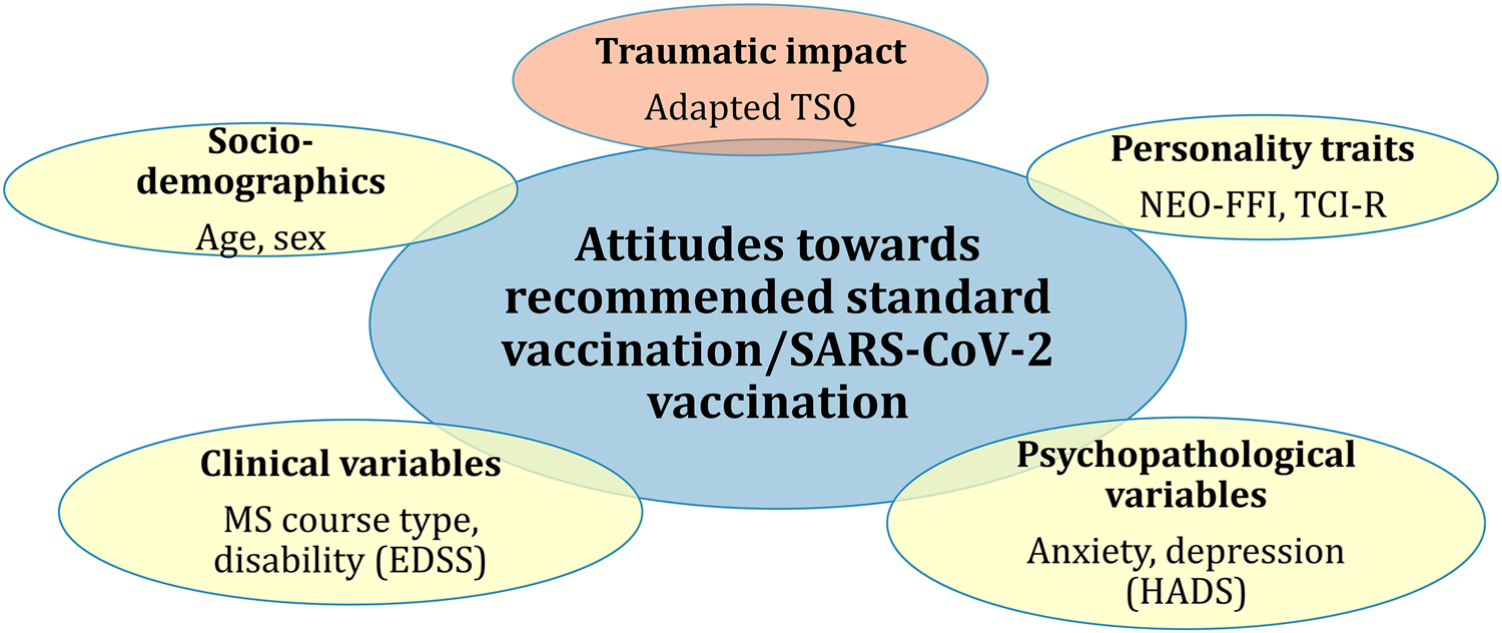
Variables to be analysed for associations with vaccination willingness in general and regarding SARS-CoV-2. *EDSS* Expanded Disability Status Scale, *HADS* Hospital Anxiety and Depression Scale, *MS* Multiple sclerosis, *NEO-FFI* NEO Five Factor Inventory; *TCI-R* Temperament and Character Inventory Revised, *TSQ* Trauma Screening Questionnaire.

Four hundred and four individuals aged ≥ 18 years diagnosed with clinically isolated syndrome (CIS) or MS according to the revised McDonald criteria^33^ have been included in a cross-sectional multi-centre study on polypharmacy and vaccination status after signing an informed consent starting in June 2019. Detailed description of the sample is incuded in the study by Heidler et al.^34^. The consecutive convenient sample was recruited at the department of neurology of Rostock’s University Medical Centre and at the neurological department of the Ecumenical Hainich Hospital in Mühlhausen/Thuringia (Germany). The patients have initially been recruited on the occasion of regular visits at the institutional MS outpatient clinics or during an inpatient stay. In total, 404 patients participated in the baseline survey (of 462 patients asked to participate). Because of the occurrence of the SARS-CoV-2 pandemic, this investigation was extended by two follow-up investigations on attitudes towards general as well as SARS-CoV-2 vaccination and on the perceived impact of federal SARS-CoV-2 safety measures in Germany in a subsample (1st follow-up: June to July 2020 before a vaccine against SARS-CoV-2 was available—N = 200, 2nd follow-up assessment: March 2021 to May 2021 after vaccines against SARS-CoV-2 were available—N = 157), see Fig. 1. There was neither any substantial dropout of participants from the baseline to the first follow-up nor to the second follow-up indicated by non-significant differences between the investigated groups on age, sex, Expanded Disability Status Scale (EDSS) score, Hospital Anxiety and Depression Scale (HADS) scores, MS course type, number of comorbid illnesses, and willingness to accept governmentally recommended vaccinations. Thus, there was no systematic loss of participants.

The study was approved by the Ethics committees of the University of Rostock (permit number A 2019-0048) and of the Physicians’ Chamber of Thuringia and conducted according to the Declaration of Helsinki.

### Data acquisition

The MS-related clinical, socio-demographic (e.g. age, sex, education and occupation), and basic vaccination-related data as well as psychopathological scores and personality data were obtained in 2019 (baseline) by a structured interview (Supplementary Document S1), a review of clinical patient records, and a clinical examination. Patients were asked to provide their vaccination card for a check of vaccination completeness based on the current recommendations by the German Standing Committee on Vaccination (STIKO) of the Robert-Koch-Institute, the German national institute for investigation and prevention of infectious diseases, and responsible for health monitoring^35^. Data on the MS course type, EDSS score^36^, disease duration, and medication was collected as well as patients’ assumption about their vaccination status and their attitudes towards vaccination in general (completed by 404 patients). Psychopathological syndrome severity was measured by the HADS (completed by 373 patients) and personality characteristics were assessed, measured by the Neo Five Factor Inventory (NEO-FFI), based on the Big-5 model of personality (completed by 373 patients), and by the Temperament and Character Inventory-revised (TCI-R, German version) derived from Cloninger’s psychobiological model of personality presented as paper-and-pencil tests^37^ (completed by 233 patients). The consequences of the pandemic and the related governmental measures were assessed by an adapted trauma questionnaire (social, psychological, and physical burden; Trauma Symptom Questionnaire [TSQ])^38^, attitudes towards standard vaccination and willingness and possibly their changes to get vaccinated against SARS-CoV-2 were collected twice during the pandemic (1st and 2nd follow-up, completed by all patients participating in the respective follow-ups).

### HADS

The HADS consists of 14 symptom-items (seven each measuring anxiety or depression) to be answered based on a four-point answer model (0–3). A clear two-factor structure was found in a factor analysis; and substantial correlations with the Beck Depression Inventory, the State-Trait Anxiety Inventory and domain scores of the Short-Form Health Survey indicated its high concurrent validity in a Spanish study of its construct validity^39^. A test–retest correlation of high effect size (r = 0.85) as well as high Cronbach’s alpha coefficients of 0.86 for both of the psychopathological syndrome scores suggested its high reliability.

### NEO-FFI

The NEO-FFI consists of 60 items, split into the five personality factors Neuroticism, Extraversion, Openness, Agreeableness, and Conscientiousness to be answered by a five—point Likert type answer model (1 = strongly disagree to 5 = strongly agree). It was tested for validity and reliability in a sample of 419 MS-patients with satisfactory internal consistency, factorial validity, and congruence between self-disclosure and third-party disclosures^40^. Cronbach’s alpha coefficients between 0.66 for Openness and 0.84 for Neuroticism or 0.71 for Oenness, Agreeableness and Conscientiousness, and 0.85 for Neuroticism were established in big samples of psychosomatic outpatients and of individuals from the general public, respectively, in Germany^41^.

### TCI-R

The TCI-R consists of 240 items divided into the seven domains described in Cloninger’s personality model (Harm Avoidance, Novelty Seeking, Reward Dependence, Persistence, Self-Directedness, Cooperativeness, and Self-Transcendence) to be answered by a true–false answer model in its German version^37^. Cronbach's alpha coefficients for the domains ranged from 0.54 to 0.83 in a general population sample, and retest reliability ranged between 0.51 and 0.74 in psychiatric patients.

### Adapted TSQ

To analyse the impact of SARS-CoV-2 pandemic (in terms of fear, burden, or changes in attitudes towards vaccination), a short questionnaire was created and presented in a telephone interview. To assess the burden caused by the SARS-CoV-2 pandemic itself and the related information in the media as well as limiting social measures, the TSQ was adapted^38^. The formulation of each item has clearly been focused on the SARS-CoV-2 pandemic and the original alternative response model was changed to a five-point scale with 0 = not at all to 4 = very much. The TSQ consists of ten symptom items (five arousal and five re-experiencing items) to assess post-traumatic stress disorder symptoms, and it is recommended to screen survivors of traumatic stress. A sum-score was calculated as indicator for burden caused by the introduced measures to limit the infection rate within the country and by living with the danger to become infected as well as suffer from a severe SARS-CoV-2 disease comorbid to the given MS.

### Statistics

Interval scaled patient data were presented by mean score and standard deviation. Ordinal or nominal scaled data are reported by percentages within the categories. Spearman Rho correlation coefficients were used to determine relationships between interval scaled data. Associations between interval scaled data and ordinal (e.g., intensity of burden) or nominal scaled data were calculated by one-way analysis of variance (ANOVA) with related post-hoc tests depending on equality of variances (equal variances: Tuckey’-b; unequal variances: Games-Howell), or by point-biserial correlation coefficients in case of a nominal scaled variable with just two categories related to an interval scaled variable. Relationships between ordinally scaled or nominally scaled data were defined by chi-squared tests or contingency coefficients, respectively. The cut-off indicating significant results was set to p = 0.050.

Since the study clearly was of an explorative nature, the alpha level was not controlled for multiple testing. All calculations were run using SPSS, version 25.

## Results

### Study population at baseline

Basic socio-demographic data and clinical information of the subsample of MS-patients included in all three assessments are presented in Table 1.

**Table 1:** Patient characteristics at baseline.

	Baseline
N (all three assessments)	157
Age (years)	49.0 ± 12.5
Disease duration (years)	12.1 ± 8.9
EDSS score	3.6 ± 2.4
Education (years)	10.5 ± 1.2
Intensity physical burden	2.5 ± 1.2
Intensity psychological burden	2.6 ± 1.2
Intensity social burden	2.4 ± 1.3
MS course type	
CIS	3.8%
RRMS	64.1%
SPMS	24.4%
PPMS	7.7%
Number of comorbid diseases	1.9 ± 1.7
DMD-treated	59.9%
Trauma score	17.6 ± 6.3

*CIS* clinically isolated syndrome, *DMD* disease modifying drug, *EDSS* expanded disability status scale, *MS* multiple sclerosis, *N* number of patients *PPMS* primary progressive MS, RRMS relapsing–remitting MS, *SPMS* secondary progressive MS.

### Indicators for attitudes towards vaccination in general

**Figure 2:**
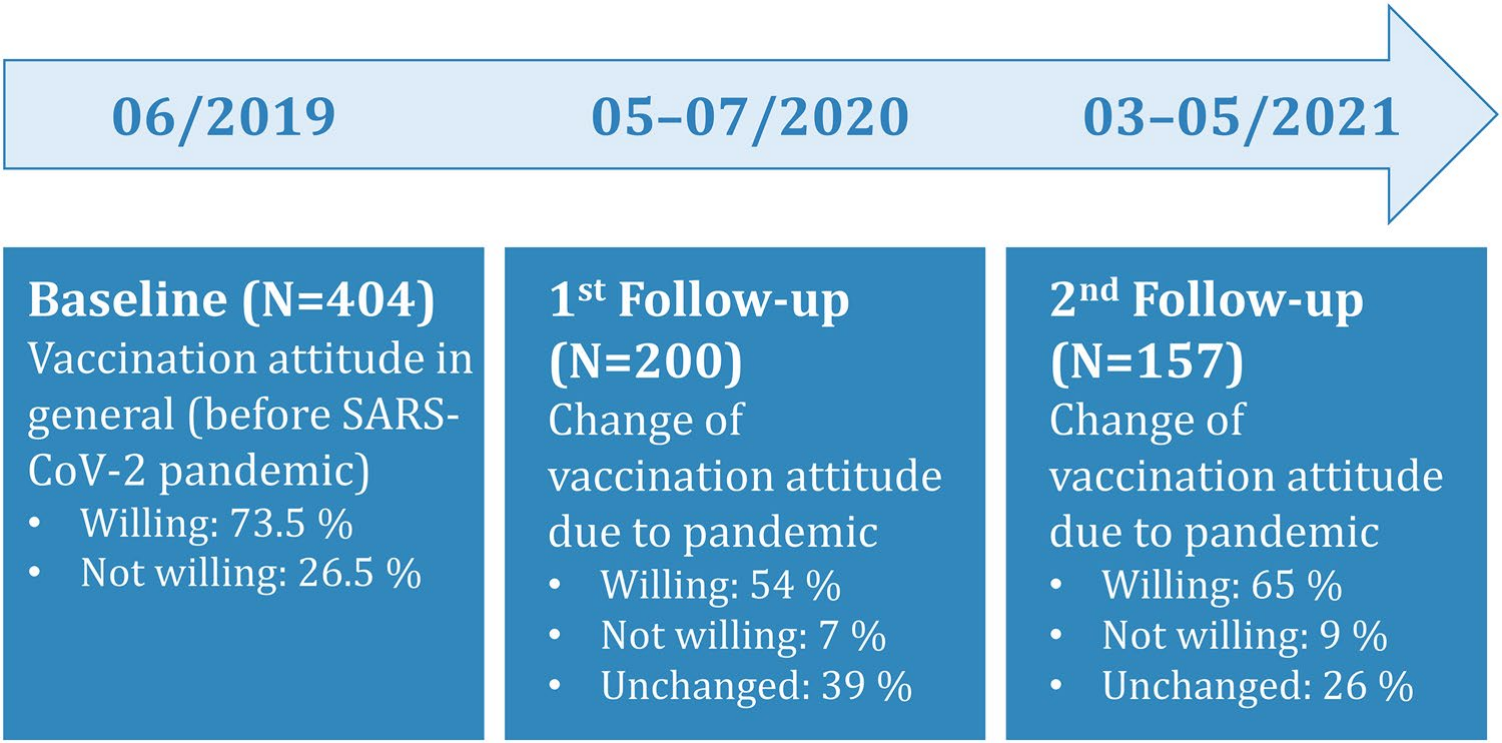
Development of general vaccination attitude before and during the course of the SARS-CoV-2 pandemic. *N* number of patients, *SARS-CoV-2* Severe acute respiratory syndrome coronavirus 2.

Over 70% of the 404 MS-patients analysed at baseline (from June 2019) indicated that they would be willing to follow the governmentally recommended vaccination program (73.5%), see Fig. 2.

Due to the discussion about the SARS-CoV-2 pandemic, 54% of the 200 patients of the 1st follow-up (June 2020 to July 2020) reported that their willingness to accept the governmentally recommended vaccination program would have been changed to agreement or that they have always been in favour of such a program. However, 7% developed or already had an anti-attitude, and 39% reported no impact of the discussions on their attitudes towards the recommended vaccinations. Regarding the SARS-CoV-2 vaccination willingness, 60% indicated that they probably or with certainty will accept a vaccination when a recommended vaccine will be available, while 23% said no or it would be unlikely, and 17% did not know yet.

At the 2nd follow-up in February/March 2021, when several SARS-CoV-2 vaccines were already approved and governmentally recommended, 65% of the 157 repeatedly investigated MS-patients indicated that their willingness to accept the governmentally recommended vaccination program would have been changed to agreement or that they always have been in favour of such a program due to the discussions about the SARS-CoV-2 pandemic. Only 9% developed or always had an anti-attitude, and 26% reported no impact of the discussions on their attitudes towards the recommended vaccinations. Regarding the SARS-CoV-2 vaccination, 61% of the patients expressed that they probably or with certainty will accept a vaccination, while 21% would not or unlikely accept such a vaccination, and 18% did not know yet.

### Association of attitude towards recommended standard vaccinations and SARS-CoV-2 vaccination

The indicated willingness to take recommended vaccinations at baseline was significantly associated to the attitude change regarding the public discussions about the SARS-CoV-2 pandemic at the 1st follow-up assessment (c^2^ [4] = 15.39; p = 0.004), but not with the attitude change between the 1st and the 2nd follow-up assessment (c^2^ [4] = 5.41; p = 0.247). During the 1st follow-up assessment, those who did not accept the recommended vaccinations at baseline (N = 107) more often developed a negative attitude towards vaccination in general due to SARS-CoV-2 discussions (16% vs. 3%), more often reported no attitude change (48% vs. 37%), and less frequently changed to a positive attitude or always have been in favour for the recommended vaccination program (36% vs. 60%) compared to MS-patients accepting recommended vaccinations at baseline.

The indicated willingness to take recommended vaccinations at baseline was significantly associated to the expressed willingness to take a SARS-CoV-2 vaccine when it would be available and recommended at the 1st follow-up assessment (c^2^ (4) = 14.59; p = 0.006), but not with the willingness to take a SARS-CoV-2 vaccine after its availability in February and March 2021 (2nd follow-up: c^2^ (4) = 5.36; p = 0.253). Significantly more willing than unwilling patients regarding the recommended standard vaccinations at baseline indicated that they would get vaccinated against SARS-CoV-2 (67% vs. 42%) at 1st follow-up, whereas standard-vaccination-unwilling patients were more likely not to get vaccinated against SARS-CoV-2 (38% vs. 17%). In both groups, there were patients who were undecided regarding their attitude towards SARS-CoV-2 vaccination at the time of the 1st follow-up (standard-vaccination willing vs. -unwilling: 16% vs. 10%).

There was a significant association in the attitude change regarding standard vaccinations due to the SARS-CoV-2 discussions (c^2^ (16) = 69.12; p < 0.001) as well as for the willingness for taking a SARS-CoV-2 vaccine (c^2^ (16) = 79.59; p < 0.001) between the 1st and 2nd follow-up assessment (see Table 2).

**Table 2:** Associations between 1st and 2nd follow-up (percentage − general vaccination attitudes/willingness for SARS-CoV-2 vaccination).

	2nd follow-up (2021)		
1st follow-up (2020)	A	B	C
A	50/56	20/19	30/25
B	7/14	36/43	57/43
C	6/9	21/13	73/78

A—Always anti or developed general negative attitudes towards vaccinations recommended/certainly not or unlikely taking SARS-CoV-2 vaccine; B—Unchanged attitudes towards recommended vaccinations/not known yet towards a SARS-CoV-2 vaccine; C—Always pro or developed positive attitudes towards recommended vaccinations/probably or with certainty taking the SARS-CoV-2 vaccine. Meaning of percentages in a field—example field A/A: 50% of those MS-patients with a negative attitude towards general vaccination recommendations during the 1st follow-up had also a negative attitude during the 2nd follow-up; 56% of those with a negative attitude towards a SARS-CoV-2 vaccination during the 1st follow-up had still a negative attitude at the 2nd follow-up.

### Determinants of attitude change towards standard vaccination and the willingness for SARS-CoV-2 vaccination

#### Socio-demographic patient characteristics (age and sex)

Age significantly positively correlated with a change in the general attitudes towards recommended vaccinations at both follow-up assessments (1st follow-up: Spearman rho = 0.21, p = 0.003; 2nd follow-up: Spearman rho = 0.19, p = 0.017) as well as with willingness to get vaccinated against SARS-CoV-2 (1st follow-up: Spearman rho = 0.27, p < 0.001; 2nd follow-up: Spearman rho = 0.19, p = 0.017), but did not with the willingness to follow recommended vaccinations at baseline (r_pbis_ = −0.09, p = 0.190).

Substantial sex differences occurred only for the willingness to get vaccinated against SARS-CoV-2 at the 2nd follow-up in 2021 (c^2^ (1) = 14.66; p = 0.005), with more female than male patients still have been undecided (26% vs. 4%), and more male than female patients were willing to take the vaccine probably or with certainty (73% vs. 54%).

#### Clinical patient characteristics

Considering MS course type, there were two significant associations with the willingness to get vaccinated against SARS-CoV-2 during the 1st follow-up (c2 [12] = 23.13; p = 0.027): significantly fewer CIS patients compared with the other disease courses were willing to be probably or definitely vaccinated against SARS-CoV-2 (36.5% [CIS] vs. 61.0 [relapsing–remitting MS − RRMS]/61.0% [secondary progressive MS − SPMS]/73.0% [primary progressive MS − PPMS]), and significantly fewer PPMS-patients compared with the other MS types reported being unlikely or certain not to get a SARS-CoV-2 vaccine (7.0% [PPMS] vs. 22.0% [SPMS]/23.0% [RRMS]/36.5% [CIS]), see Table 3. Even though age and MS course type were found substantially interrelated (Kendall's tau-b = 0.30; p < 0.001), age was substantially more associated with the willingness to take an anti-SARS-CoV2 vaccine (Kendall's tau-b = 0.15; p = 0.017) than the MS-course type (Kendall's tau-b = 0.07; p = 0.360) indicating that age and possibly the related personality maturity are of higher impact than MS-course type. There were no significant associations between the SARS-CoV-2 vaccination willingness or the attitude change regarding recommended standard vaccinations with EDSS, number of comorbidities, number of drugs taken, or number of DMD switches at any examination period.

**Table 3:** Association between MS course type and willingness to take SARS-CoV-2 vaccine (percentage within course type groups at 1st follow-up/2nd follow-up).

	A	B	C	N
CIS	36.5/0.0	27.0/33.3	36.5/67.0	11/6
RRMS	23.0/25.0	16.0/20.0	61.0/55.0	128/100
SPMS	22.0/14.0	17.0/13.0	61.0/73.0	40/38
PPMS	7.0/27.0	20.0/9.0	73.0/64.0	15/11

A—Certainly not or unlikely taking a SARS-CoV-2 vaccine; B—Not known yet; C—Probably or with certainty taking a SARS-CoV-2 vaccine; *CIS* clinically isolated syndrome, *MS* multiple sclerosis, *PPMS* Primary progressive MS, *N* total number of patients, *RRMS* relapsing–remitting MS, *SPMS* Secondary progressive MS. Meaning of percentages in a field − example field RRMS/A: 23.0% of RRMS-patients at the 1st follow-up and 25.0% at the 2nd follow-up were certainly not or unlikely taking a SARS-CoV-2 vaccine.

#### Anxiety and depression

Anxiety severity (HADS-A score) at baseline was not substantially related to any of the attitudes towards SARS-CoV-2 vaccination indicators at any of the follow-up assessments. Depression severity (HADS-D score) significantly negatively correlated with the general attitude change towards recommended standard vaccinations due to the public SARS-CoV-2 pandemic discussion (1st follow-up: Spearman rho = −0.14, p = 0.049; 2nd follow-up: Spearman rho = −0.29, p < 0.001) with the more severely depressed the MS-patients were at baseline, the less the attitude change at both follow-up assessments. The trauma-score was substantially positively associated with the willingness to take a SARS-CoV-2 vaccine within the 1st follow-up in 2020 (1st Spearman rho = 0.15, p = 0.039) with the higher the trauma score was the more the patients were willing to get vaccinated against SARS-CoV-2 virus, while it was significantly negatively related to the attitude change towards general vaccination within the 2nd follow-up with low effect size (2_nd_ Spearman rho = −0.24, p = 0.003).

#### Personality characteristics

**Figure 3:**
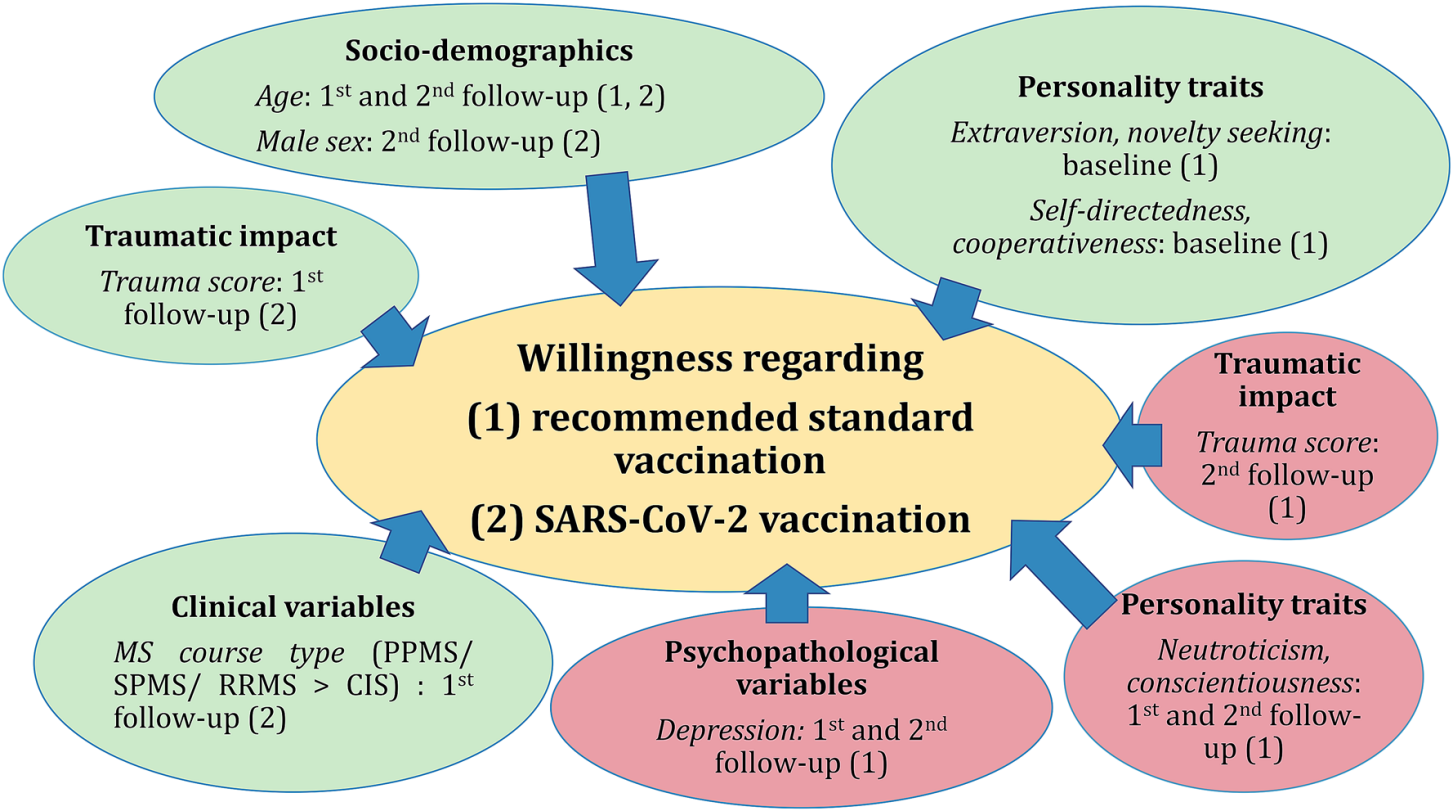
Patient characteristics associated with the willingness regarding recommended standard vaccinations and SARS-CoV-2 vaccinations. The coloured elements show the significant positive (green) or negative (red) associations of socio-demographic, clinical variables, personality traits, psychopathological variables, as well as traumatic impact with the willingness regarding (1) recommended standard vaccinations and (2) SARS-CoV-2 vaccination during the baseline investigation and the two follow-ups. *CIS* Clinically isolated syndrome, *MS* Multiple Sclerosis, *PPMS* Primary progressive MS, *RRMS* Relapsing remitting MS, *SARS-CoV-2* severe acute respiratory syndrome coronavirus 2, *SPMS* Secondary progressive MS.

The willingness to comply with the governmentally recommended vaccinations at baseline was significantly correlated with the Extraversion score (r_pbis_ = 0.20; p = 0.007) and the Novelty Seeking score (r_pbis_ = 0.18; p = 0.017), with those accepting the recommendations scoring higher in both variables, see Fig. 3. The higher the Neuroticism score (1st follow-up: Spearman rho = −0.14, p = 0.049; 2nd follow-up: Spearman rho = −0.40, p < 0.001) and the Conscientiousness score (1st Spearman rho = −0.04, p = 0.594; 2nd Spearman rho = −0.21, p = 0.009) of the NEO-FFI, the more dismissive was the change of attitudes towards vaccination in general at both follow-up assessments. Furthermore, the higher the MS-patients scored for Self-Directedness (1st follow-up: Spearman rho = 0.07, p = 0.343; 2nd Spearman rho = 0.20, p = 0.018) and for Cooperativeness (1st follow-up: Spearman rho = 0.10, p = 0.194; 2nd follow-up: Spearman rho = 0.20, p = 0.018) at baseline (TCI-R), the more they favourably changed their attitudes towards vaccination in general at both follow-up assessments.

All other scores for personality characteristics did not significantly correlate with any attitude or willingness for getting a SARS-CoV-2 vaccine.

## Discussion

The present longitudinal, prospective multi-centre-study investigated German MS-patients’ attitudes towards a SARS-CoV-2 vaccination and governmental measures before and after the 1st SARS-CoV-2 pandemic wave and within the 3rd wave in Germany based on socio-demographic, clinical, and personality variables. A high reported acceptance to comply with governmentally recommended vaccinations from the STIKO of 75% was found in the baseline investigation before the SARS-CoV-2 pandemic started.

The 1st follow-up was carried-out during or shortly after the 1st wave of the SARS-CoV-2 pandemic (June 2020). No vaccine against SARS-CoV-2 was available at this time. This 1st follow-up showed a major acceptance of a vaccination against SARS-CoV-2: 60.5% of the patients were willing to get a vaccine, 17.0% were undecided, and 22.5% were against it^34^. Although the majority of MS-patients analysed in the present study showed willingness to get vaccinated against SARS-CoV-2 at both follow-ups (1st: 60%, 2nd: 61%), a substantial proportion had concerns and were undecided or opposed to vaccination. This is lower than in MS-patients in the U.S.A. with 66.0–70.1%^24,42^. A higher rate of vaccination endorsement (80.9%) was found in a small Portuguese study^25^. In a comparable survey period to our first follow-up (June to July 2020), 70% of participants of a real time survey as a part of the German Socio-Economic Panel were willing to become vaccinated against SARS-CoV-2^43^. These results match another online survey from April 2020 in which 73.9% of 7664 participants from seven European countries (United Kingdom, Germany, Netherlands, Portugal, France, Denmark and Italy) reported that they would be in favour of getting vaccinated against SARS-CoV-2, while 18.9% were undecided and 7.2% were unwilling to get vaccinated^30^. Consequently, the vaccination willingness in our surveyed MS patients at the time of the 1st follow-up was about 10% lower than in the general population.

A general vaccination hesitancy can have many causes^44^. The perceived absence of feeling danger can be due to various thoughts. Perceiving a lack of usefulness of the vaccination as well as the feeling that the situation is not serious may be one subjective assumption. The personal risk may be considered as low, which can cause a lower vaccination willingness. A high awareness for risk perception like, the vaccine has not been tested adequately or has been tested too quickly after insufficient research, can lead to a refusal of a vaccination. The media documentation of the economic goals of vaccine manufacturers can contribute to patient uncertainty^45,46^. The fear of affecting the personal health negatively by a vaccine can additionally cause a refusal of vaccination, in terms of being enabled to father healthy children any longer; to unfavourably impact on a chronic illness; or to expose the body to a foreign, unfamiliar substance at all^44^. The lack of knowledge or belief that being vaccinated protects others as well as a low social pressure are important factors as well as the conviction that others also refuse the vaccination. The general interaction with the health care system like the frequency of consulting physicians or getting direct recommendations could be additional decisive factors. However, our findings suggest the substantial role of general, pre-pandemic attitudes towards recommended vaccinations regarding the willingness to get vaccinated against SARS-CoV-2. The results pointed to a high stability of vaccination willingness independent on the course of the pandemic and on the discussion of this topic in the media.

An important socio-demographic variable associated with SARS-CoV-2 vaccination willingness was age. The older the patients, the higher their acceptance to get a SARS-CoV-2 vaccine, which was also found in other studies in the general U.S.-population^47^. A higher perception of risks, due to a longer time of experience, could be one reason. Several other socio-demographic and clinical characteristics like sex, number of comorbid illnesses (like in an Indian study^48^), medication, or a high disability level had no substantial impact on vaccination willingness in our study. However, a sex difference was found in a study from Israel concerning vaccination attitudes in the younger population, in contrast to our data. Females were much more sceptical regarding an immediate vaccination against SARS-CoV-2 than males in the Israeli study (vaccination willingness: 12.0–13.6% vs. 23.1–27.3%)^49^.

In our study, several psychological factors were found as important influences on a decision about getting vaccinated. Attitudes towards vaccination and future vaccination behaviour correlated with factors regarding mental health and personality characteristics. These included comorbidity with depression, traumatic burden, and increased Neuroticism. Poor evidence is available regarding the plights of MS-patients during the SARS-CoV-2 pandemic. In three studies, participants from pre-pandemic investigations were re-assessed to determine, what type of stress changed disorders within studied intervals. Mixed results suggested little or no change in anxiety or depression^50^. Another investigation revealed a negative correlation to vaccination readiness in depression^51^. Even though a close association between mental health and chronic diseases was reported in several studies, we could only determine this for depression^52,53^. The more depressed the MS-patients were, the lower the indicated change of attitudes towards the generally recommended vaccinations due the pandemic and the discussion in the media at both follow-up investigations of our study. Furthermore, the more intensively traumatized by the pandemic, the governmental measures against the pandemic and the related discussion in the media, the MS-patients were open to accept a SARS-CoV-2 vaccine at the 1st follow-up, but not at the 2nd follow-up. When individuals have been traumatized by the pandemic, it might have been a meaningful consequence to do everything to avoid an infection and to do, what is possible, to avoid a serious course of COVID-19. A vaccination currently seems to be the only recommended opportunity. The difference of the associations between the two follow-ups might be caused by the long-lasting course of the pandemic and by the increasingly upcoming negative arguments on the conduct of the studies, the decreasing vaccine efficacy over time and the various side-effect potential as well as management of the several vaccines in the media^54–60^.

Furthermore, the more outgoing, energetic, and assertive (high on Extraversion) and the more exploratory, curious, and enthusiastic (high on Novelty Seeking; Cloninger et al.^37^) the MS-patients were, the more likely they reported their willingness to accept recommended vaccinations at the baseline investigation. The more organized and the more diligently working toward achieving goals (high on Conscientiousness) and the more anxious, angrily hostile, depressed, and vulnerable (high on Neuroticism) the MS-patients were, the less they changed their attitudes towards recommended vaccinations due to the pandemic at both follow-up assessments. This indicates that different personality characteristics can cause stability of attitudes. On the one hand, stability could be due to goal directed behaviour and, on the other hand, anxiety, depressed mood and vulnerability could lead to an avoidance of change. However, the more responsible, reliable, and goal-oriented (high on Self-Directedness) and the more empathic, supportive, and principled (high on Cooperativeness) the MS-patients were, the more their attitudes towards governmentally recommended vaccinations changed in the direction of acceptance due to the pandemic between baseline assessment and both follow-up assessments. According to our data, highly mature individuals seem to accept and follow recommended vaccinations more often than less mature individuals.

The generalizability of our findings is somewhat limited by the included sample size, the drop-out rate of participants from baseline to the 2nd follow-up (however without a systematic loss to follow-up ) and the lack of a control group of people without MS. The validity of the data might be limited since the follow-up data exclusively were patient-reported. However, it represents one of the rare studies to explore SARS-CoV-2 and general vaccination willingness among MS-patients, and, according to our best knowledge, it is so far the only one with two follow-up assessments. Furthermore, this represents the 1st investigation recognising associations between personality characteristics and psychopathological background variables with willingness of vaccination against SARS-CoV-2.

In summary, our present study suggested that about two thirds of German MS-patients analysed were willing to receive a SARS-CoV-2 vaccine. Important variables associated with vaccination willingness, reported in a previous study^34^ were socio-demographic variables such as higher age and personality characteristics such as Novelty Seeking as well as Extraversion. Changes to pro-vaccination attitudes during the follow-ups were seen in individuals with higher scores in Self-Directedness and Cooperativeness. Factors that were continuously negatively correlated to the acceptance of a vaccination against SARS-CoV-2 were depression, Neuroticism, and Conscientiousness. Personality characteristics and their impact on vaccination settings were investigated the 1st time.

Our study underlines the importance of the factors related to SARS-CoV-2 vaccination acceptance, which can be used for personalized discussions with vaccine-insecure MS-patients. Besides concerns about vaccination in general and objections against the novel type of nucleic acid-based vaccines against SARS-Cov-2, certain personality characteristics as well as psychopathological conditions, depression in particular, should be considered by doctors when talking to MS-patients about getting vaccinated. It is a big challenge to reach much higher vaccination rates than currently achievable. In this context, the question for future studies is whether the availability and approval of other types of vaccines against SARS-CoV-2, such as inactivated vaccines (e.g. Novavax [NVX-CoV 2373] or Valneva [VLA 2001]), will fundamentally change the willingness to become vaccinated.

## Supplementary Information

Supplementary Information.

## Data Availability

The datasets generated during and/or analysed during the current study are available from the corresponding author on reasonable request.
