# Supplementary material for: Vaccination willingness in association with personality traits in patients with multiple sclerosis in the course of SARS-CoV-2 pandemic
Source: Sci Rep. 2022 Sep 7;12:15147. doi: 10.1038/s41598-022-18912-3 (PMC9449937; doi:10.1038/s41598-022-18912-3)
Supplement: Supplementary file 1 — Supplementary Information. [file 41598_2022_18912_MOESM1_ESM.pdf]

Supplementary document S1: Structured interview

Basic questionnaire – 2019

Sociodemographic

Birth date: _____years

Sex: ☐ female
☐ male

Partnership status: ☐ single living
☐ partnership

Current job situation:
☐ in training
☐ studies
☐ employment: ☐ full-time ☐ part-time
☐ unemployment
☐ pension: ☐ due to old age ☐ due to disability
☐ other

School years (at most until A-levels): _____years

Educational level: ☐ no training
☐ skilled worker
☐ technical college
☐ university

Place of residence:
____
☐ rural community (until 5000 inhabitants)
☐ provincial town (>5000-20000 inhabitants)
☐ medium-sized town (>20000-100000 inhabitants)
☐ city (>100000 inhabitants)

Number of children: ____

Number of siblings: ____

Clinical-neurological

Current pregnancy: ☐ no
☐ yes

Pregnancy planned in the near future: ☐ no
☐ yes

Number and kind of comorbidities:

Disease course: ☐ clinically isolated syndrome (CIS)
☐ relapsing-remitting multiple sclerosis (RRMS)
☐ secondary progressive multiple sclerosis (SPMS)
☐ primary progressive multiple sclerosis (PPMS)
☐ undefined

Clinical care: ☐ outpatient
☐ inpatient

Date of initial CIS/MS diagnosis (month/year): ____

Current Expanded Disability Status Scale (EDSS) Score: ____

Vaccination

Vaccination card available: ☐ no
☐ yes

Did you have a vaccination consultation last year? ☐ no
☐ yes

When was the last time you dealt with the topic of "vaccination"?
☐ last week
☐ last month
☐ last six months
☐ last year
☐ more than a year ago

Are you aware that government recommendations for immunizations exist?
☐ no
☐ yes

Would you be willing to get all officially recommended vaccinations?
☐ no
☐ yes

SARS CoV-2 questionnaire 2020 (1st follow-up)

We have all been affected in the past weeks and are still affected in our lives due to the SARS CoV-2 pandemic and the behavioral restrictions instructed to protect the population or also due to our own experience. We would like to ask you to answer a few questions to find out how you, as an MS patient, have experienced and coped with this situation over the past eight weeks.

1. Has MS changed in the last three months				**(Please check)**
a) in the form of relapses?	**Yes**	☐	**No**	☐
b) in the form of progression ?	**Yes**	☐	**No**	☐
2. Did you have any other illnesses in the last three months?	**Yes**	☐	**No**	☐
	**If yes, which?**			

Please answer the following questions according to the levels:	**Not at all**	**Almost not**	**Not sure**	**Strong**	**Very strong**
3. How much psychological burden did you feel during that time?	☐	☐	☐	☐	☐
4. How much physical burden did you feel during that time?	☐	☐	☐	☐	☐
1. How much social burden did you feel during that time?	☐	☐	☐	☐	☐
2. Did any distressing thoughts or memories related to the SARS CoV-2 pandemic come to mind?	☐	☐	☐	☐	☐
3. Did you experience distressing dreams related to the SARS CoV-2 pandemic?	☐	☐	☐	☐	☐
4. Did you act or feel like this was always going to happen again?	☐	☐	☐	☐	☐
5. Were you shocked when you were reminded of this?	☐	☐	☐	☐	☐
6. Did you feel any physical reactions (such as rapid heartbeat, abdominal pain)?	☐	☐	☐	☐	☐

	Not at all	Almost not	Not sure	Strong	Very strong
7. Did you experience difficulty falling asleep or staying asleep?	☐	☐	☐	☐	☐
8. Did you experience increased irritability or outbursts of anger?	☐	☐	☐	☐	☐
9. Did you have difficulty in concentrating?	☐	☐	☐	☐	☐
10. Did you pay increased attention to possible dangers to you or others?	☐	☐	☐	☐	☐
11. Did you feel jumpier or startled more quickly than usual when something unexpected happened?	☐	☐	☐	☐	☐

12. Since the impact of the SARS CoV-2 pandemic began, have your attitudes changed regarding the SARS CoV-2 issue? (Please check)	**Yes**	☐	**No**	☐

13. Today, do you experience most of the measures ordered as appropriate, even in retrospect?	**Not appropriate at all**	**Somewhat inappropriate**	**Unsure**	**Somewhat appropriate**	**Completely appropriate**
	☐	☐	☐	☐	☐

14. Has your general attitude toward standard vaccination changed as a result of the discussions surrounding the SARS CoV-2 pandemic?	**My anti attitude was strengthened**	**Have developed an anti attitude**	**Unchanged**	**Accept now a general vaccination program**	**Was always in favor of a vaccination program**
	☐	☐	☐	☐	☐

15. What do you think today? If an effective vaccine against SARS CoV-2 is found and the Standing Committee in Vaccination recommends this vaccination, will you get vaccinated?	**Very unlikely**	**Unlikely**	**Unsure**	**Likely**	**For sure**
	☐	☐	☐	☐	☐

Questions 4 – 13 adapted from Trauma Screening Questionnaire (TSQ) Brewin et al. (2002) Br. J. Psychiatry

SARS CoV-2 questionnaire 2021 (2nd follow-up)

We have all been affected in the past weeks and are still affected in our lives due to the SARS CoV-2 pandemic and the behavioral restrictions instructed to protect the population or also due to our own experience. We would like to ask you to answer a few questions to find out how you, as an MS patient, have experienced and coped with this situation over the past eight weeks.

1. Has MS changed in the last three months					**(Please check)**
a) in the form of relapses?	**Yes**	☐	**No**	☐	
b) in the form of progression ?	**Yes**	☐	**No**	☐	
2. Did you have any other illnesses in the last three months?	**Yes**	☐	**No**	☐	
	**If yes, which?**				
3. Did you have any vaccinations (of any kind) in the last three months?	**Yes**	☐	**No**	☐	
	**If yes, which?**				
	**Did you experience health issues during/after vaccination?**	**No**	**Yes, mild vaccine reactions**	**Yes, vaccine diseases / complica-tions**	
		☐	☐	☐	
4. Were you ill with coronavirus (SARS-CoV-2) or did you have a positive antigen test?	**Yes**	☐	**No**	☐	
	**If yes, did you experience**	**No symptoms**	**Mild/moderate symptoms being treated on outpatient basis**	**Severe symptoms requiring inpatient treatment**	
		☐	☐	☐	

Please answer the following questions according to the levels:	**Not at all**	**Almost not**	**Not sure**	**Strong**	**Very strong**
5. How much psychological burden did you feel during the last three months?	☐	☐	☐	☐	☐
6. How much physical burden did you feel during the last three months?	☐	☐	☐	☐	☐
7. How much social burden did you feel during the last three months?	☐	☐	☐	☐	☐
8. Did any distressing thoughts or memories related to the SARS CoV-2 pandemic come to mind?	☐	☐	☐	☐	☐
9. Did you experience distressing dreams related to the SARS CoV-2 pandemic?	☐	☐	☐	☐	☐
10. Did you act or feel like this was always going to happen again?	☐	☐	☐	☐	☐
11. Were you shocked when you were reminded of this?	☐	☐	☐	☐	☐
12. Did you feel any physical reactions (such as rapid heartbeat, abdominal pain)?	☐	☐	☐	☐	☐
	**Not at all**	**Almost not**	**Not sure**	**Strong**	**Very strong**
13. Did you experience difficulty falling asleep or staying asleep?	☐	☐	☐	☐	☐
14. Did you experience increased irritability or outbursts of anger?	☐	☐	☐	☐	☐
15. Did you have difficulty in concentrating?	☐	☐	☐	☐	☐
16. Did you pay increased attention to possible dangers to you or others?	☐	☐	☐	☐	☐
17. Did you feel jumpier or startled more quickly than usual when something unexpected happened?	☐	☐	☐	☐	☐
18. Since the impact of the SARS CoV-2 pandemic began, have your attitudes changed regarding the SARS CoV-2 issue? (Please check)	**Yes**	☐	**No**	☐	

19. Today, do you experience most of the measures ordered as appropriate, even in retrospect?	**Not appropriate at all**	**Somewhat inappropriate**	**Unsure**	**Somewhat appropriate**	**Completely appropriate**
	☐	☐	☐	☐	☐
20. Has your general attitude toward standard vaccination changed as a result of the discussions surrounding the SARS CoV-2 pandemic since the last survey (summer 2020)?	**1. My anti attitude was strengthened**	**2. Have developed an anti attitude**	**3. Unchanged**	**4. Accept now a general vaccination program**	**5. Was always in favor of a vaccination program**
	☐	☐	☐	☐	☐
	**If 1., 2. or 4., what was the main reason?**				
21. What do you think today? If an effective vaccine against SARS CoV-2 is found and the Standing Committee in Vaccination recommends this vaccination, will you get vaccinated?	**Very unlikely**	**Unlikely**	**Unsure**	**Likely**	**For sure**
	☐	☐	☐	☐	☐
22. Would you prefer a certain vaccine?	**Yes**	☐		**No**	☐
	**If yes, due to**	**Efficacy**	**Tolerability**	**Availability**	**Other**
		☐	☐	☐	☐

Questions 6 – 15 adapted from Trauma Screening Questionnaire (TSQ) Brewin et al. (2002) Br. J. Psychiatry
